# Associations of dietary selenium intake with the risk of chronic diseases and mortality in US adults

**DOI:** 10.3389/fnut.2024.1363299

**Published:** 2024-06-24

**Authors:** Yuchen Zhang, Shixin Meng, Yuexin Yu, Liangwen Bi, Jihong Tian, Lizhen Zhang

**Affiliations:** ^1^The Second Affiliated Hospital of Nanjing Medical University, Nanjing, China; ^2^The Basic Medical Sciences College of Nanjing Medical University, Nanjing, China; ^3^Shanghai General Hospital, Shanghai, China; ^4^Shanghai Jiao Tong University School of Medicine, Shanghai, China

**Keywords:** selenium intake, cardiovascular disease, diabetes mellitus, mortality, NHANES

## Abstract

**Objective:**

Selenium is an essential micronutrient and a type of dietary antioxidant. This study aimed to investigate the associations of dietary selenium intake with the risk of human chronic disease [cardiovascular disease (CVD), diabetes mellitus (DM), and cancer] and mortality among US general adults.

**Methods:**

The dietary and demographic data in this study were collected from the National Health and Nutrition Examination Survey (NHANES) from 2007 to 2018. Death outcomes were determined by associating with the National Death Index (NDI) records as of December 31, 2019. Logistic regression analyses were used to investigate the relationship of selenium intake with the risk of CVD, DM, and cancer. The effect of dietary selenium on all-cause and disease-specific mortality was estimated with restricted cubic spline (RCS) curves based on the univariate and multivariate Cox proportional hazard models.

**Results:**

Among the 25,801 participants, dietary selenium intake was divided into quintiles (Q1–Q5). After covariate adjustment, the results showed that the participants with higher quintiles (Q4 and Q5) of selenium intake tended to have a low risk of CVD (OR = 0.97, 95% CI: 0.96, 0.99; OR = 0.98, 95% CI: 0.97, 1.00, respectively). Moreover, the RCS curves showed a significant nonlinear association between selenium intake and the risk of all-cause (with a HR of 0.82, 95% CI: 0.68, 0.99) and DM-specific mortality (with the lowest HR of 0.30; 95% CI, 0.12–0.75). Furthermore, we conducted a subgroup analysis and found a negative correlation between the highest quartile of selenium intake and all-cause mortality among participants aged 50 and above (HR = 0.75, 95% CI: 0.60–0.93, *p* = 0.009).

**Conclusion:**

Our results indicated that a moderate dietary selenium supplement decreased the risk of CVD and displayed a nonlinear trend in association with the risk of all-cause and DM-specific mortality among US adults. In addition, we found that participants aged 50 and older may benefit from higher selenium intake. However, these findings still need to be confirmed through further mechanism exploration.

## Introduction

Non-communicable chronic diseases (NCDs) mainly include CVD, DM and cancer, which are the leading causes of premature death and harm to human health (1). Numerous studies have found that excessive accumulation of reactive oxygen species (ROS) in the human body is an important risk factor for promoting the onset of chronic diseases and endangering human health (2). ROS originate from molecular oxygen, which typically includes superoxide (O2·–), hydrogen peroxide (H_2_O_2_), and hydroxyl radical (·OH). Overproduction of ROS disrupts the normal cellular biological processes by changing the activity of various signaling pathways, such as NRF2–KEAP1, NF-κB, and AMPK, and mitochondrial signaling pathways, which can cause fatal damage to cells (3).

Previous studies have identified oxidative stress as a risk factor for the development of some NCDs, such as CVD, DM, and cancer. Atherosclerosis caused by oxidative stress and chronic inflammatory cascade reaction is the most important etiology of CVD, as reported by Macvanin et al. (4). Another study indicated that one of the reasons for insulin resistance may be the destruction of pancreatic β-cells caused by oxidative stress. Furthermore, excessive ROS production plays a crucial role in the pathophysiology of DM, leading to cell damage and disease progression (5). Chronic oxidative stress is a common condition in patients with cancers. Excessive ROS production can induce oxidative damage and inflammation, which, in turn, lead to the progression of various kinds of cancer, including hepatocellular carcinoma, lung cancer, and colon cancer (6–8).

Various treatment methods have been continuously proposed to reduce the global disease burden and human mortality rate. In addition to medication, dietary intervention is gradually being recognized as a reliable treatment strategy to prevent the occurrence of chronic diseases and prolong human survival (9). Antioxidants can limit the adverse effects (oxidative stress and chronic inflammation) caused by ROS accumulation (10). It is considered that insufficient consumption of antioxidant-rich foods, such as vegetables and fruits, is closely associated with the occurrence of NCDs, especially CVD, DM, and cancer (11, 12). Dietary antioxidants are mainly composed of vitamin A, vitamin C, vitamin E, carotenoids, zinc, and selenium (13). Related studies have found that a high-level dietary intake of vitamin A, vitamin E, and carotenoids is associated with a lower all-cause mortality compared with a low-level intake of these micronutrients (14). These studies may provide individuals with a safe and convenient choice, namely, by having an adequate dietary antioxidant intake, the occurrence of NCDs and the risk of all-cause and disease-specific mortality can be reduced.

Selenium is a type of dietary antioxidant. Chen’s et al. (15) study showed that an appropriate concentration of selenium (6 mg/L nanoselenium) can enhance the antioxidant activity of soy sauce. As an essential micronutrient, selenium is generally considered to play an important role in maintaining human health. A selenium supplement (nanoselenium) has been shown to improve the health of various species (16). It has been proved that organic selenium is characterized by higher absorption, better antioxidant properties, and lower toxicity to the human body (17). However, even though both organic and inorganic selenium exists within our daily dietary, the phenomenon of imbalanced dietary nutritional intake still exists (18). Insufficient consumption of grains and animal foods leads to selenium deficiency, as the daily intake of selenium for a normal adult is approximately 75 ± 1 μg (19). Notably, Bama Yao Autonomous County is famous for the longevity of its residents. Li et al. (20) proved that a high level of selenium intake (82.54 μg/day) is one of the main factors for maintaining the normal physical function and longevity of the older adults in Bama Yao County. Several studies have shown that selenium deficiency increases the risk of metabolic disorders, such as dyslipidemia, abnormal glucose metabolism, and thyroid hormone dysfunction (21), through oxidative stress and abnormal activation of the PI3K/Akt signaling pathway (22). Moreover, Yildirim et al. (23) showed that selenium can prevent oxidative stress-related damage in acrylamide-induced testicular toxicity in rats. In another study, Ibrahim et al. (24) indicated that binaphthyl diselenide could significantly restore the defects of antioxidant defense mechanisms and exert anti-ulcer activity against ethanol-induced gastric injury.

However, few studies are dedicated to elucidating the harm of selenium deficiency in the US population. Therefore, it is critical to explore the effects of dietary selenium intake on human health. In this study, we systematically analyzed the association of selenium intake with the occurrence and prognosis of CVD, DM, and cancer, aiming to provide a theoretical foundation for a moderate selenium supplement in daily diet.

## Materials and methods

This is a large sample retrospective study that include 25,801 participates, with information sourced from NHANES 2007–2018. NHANES is a comprehensive cross-sectional survey conducted by the Centers for Disease Control and Prevention (CDC) in all 50 states and the District of Columbia in the United States. NHANES began in the 1980s and its data has been updated every 2 years since the end of the 20th century. NHANES database contain a large amount of survey questionnaire data, biological samples measurement indicators, physical examination markers and other content (25, 26). Detailed information and method of application can be obtained from the website: https://www.cdc.gov/nchs/nhanes/index.htm. The survival status and cause of death in NHANES can be determined by matching National Death Index (NDI) records (27).

### Study design

**Figure 1:**
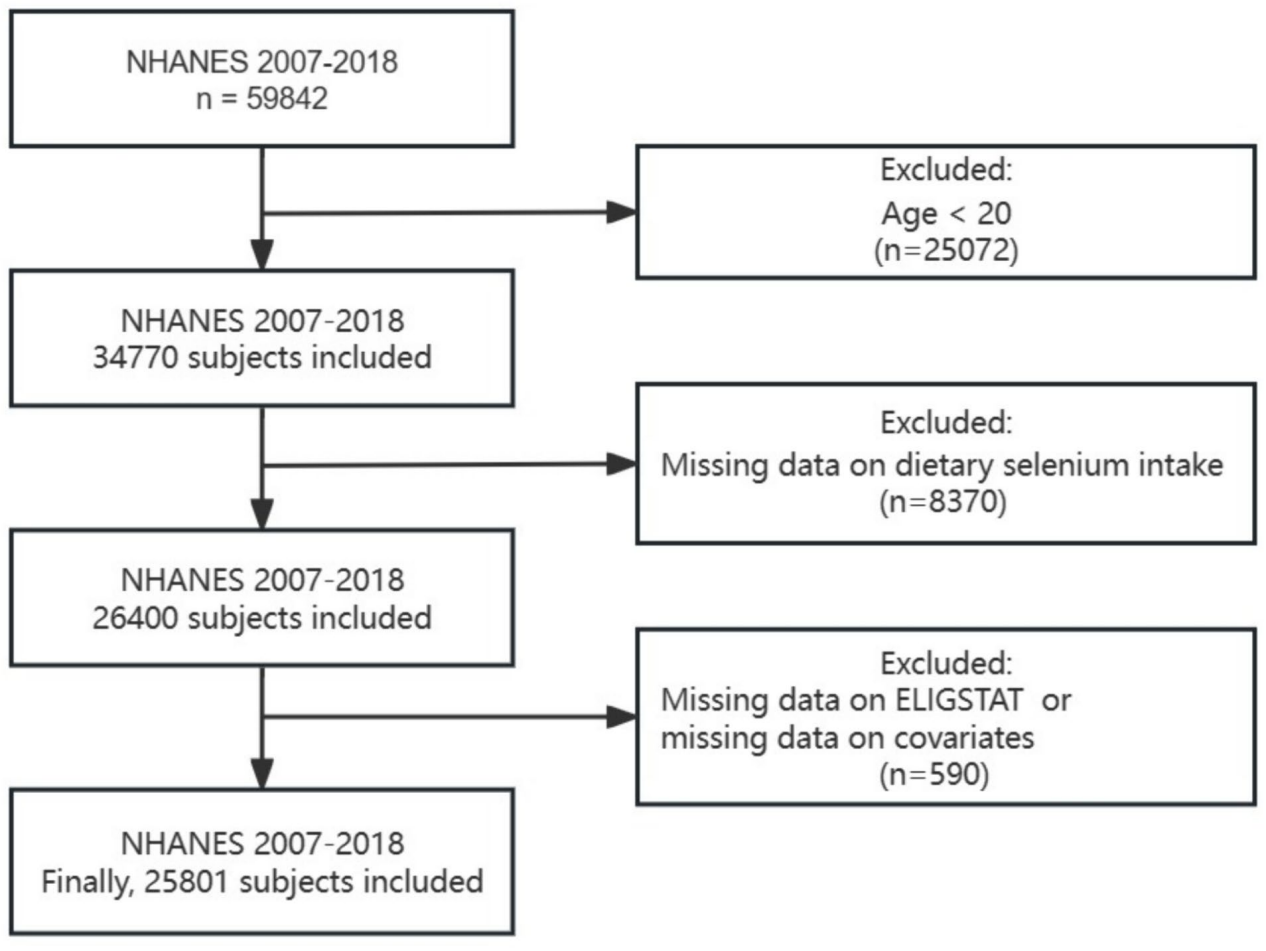
Flow chart of the sample selection in this study.

Flow chart of this study is shown in Figure 1. We utilized dietary and demographic information from NHANES 2007–2018 (*N* = 59,842). In this analysis, CVD is defined as meeting any of the following criteria: (1) Ever told had congestive heart failure; (2) Ever told you had coronary heart disease; (3) Ever told you had angina/angina pectoris; (4) Ever told you had heart attack; (5) Ever told you had a stroke. TDM is defined as satisfy the questionnaire: (1) doctor told you have diabetes, or (2) taking insulin now. And the population who answered yes in the questionnaire: ever told you had cancer or malignancy is considered as cancer patients. Participants aged 20 years or above were included in this study (*N* = 36,580). Individuals who missed selenium intake information in their diet and dietary supplements, without important covariate, and those with unavailable follow-up data on survival status were excluded. Ultimately, our analysis included a total of 25,801 participants.

### Measurement of selenium intake

The information on selenium intake in diet and dietary supplements assembled from two 24-h interviews. The first dietary recall interview was personally collected at the Mobile Examination Center (MEC), and the second interview was collected over the telephone 3 to 10 days later. In this analysis, total intake of selenium is derived from the sum of dietary and dietary supplement intake. The average dietary selenium intake was calculated through two 24-h dietary recalls, and the first 24-h value will be used instead of the average value if information is unavailable during the second 24-h recall.

### Assessment of covariates

We organized and analyzed the covariates of the included population. Based on previous researches, the covariates involved in this study are as follows: age, gender (males and females), race (Mexican American, Other Hispanic, Non-Hispanic White, Non-Hispanic Black and Other), education level (High school graduate or above), family poverty income ratio (<1.3; 1.3–3.5; ≥3.5), smoke status (current smoker, former smoker and never smoker), drink status (drinker and never drinker) and BMI (Underweight, Normal, Overweight and Obese). BMI was calculated as weight (kg)/[height(m)]^2^.

### Survival analysis

The outcome information of participants is recorded in National Death Index (NDI) and linked to NHANES through SEQN. The median follow-up time of this study was 81 months. All-cause mortality includes deaths caused by any reason during the follow-up period. Specific disease related deaths were determined using ICD-10. CVD-related death was represented as ICD 054-068, cancer-related death was represented as ICD 019-043 and T2DM-related death was represented as ICD 046. There were total of 2,829 CVD-related mortality, 2,578 cancer-related mortality and 3,417 T2DM-related mortality.

### Statistical analysis

We extracted data from six NHANES cycles (2007–2018) and preformed all analyses in accordance with the complex multistage probability design offered by NHANES officials. The characteristics of participants were exhibited using survey-weighted means and proportions. A total of selenium intake was divided into quintiles. Wilcoxon rank sum test and Kruskal–Wallis test were used to compare the baseline characteristic differences between populations with different quintiles of selenium intake. We performed survey-weighted logistic regression model to illustrate the association between selenium intake and chronic disease, and survey-weighted cox proportional hazards model was used to investigate the role of dietary selenium intake in survival outcomes. Results of the model were presented as odds ratio (OR) or hazard ratio (HR), and 95% confidence interval (CI) were calculated to estimate the range of values for variables. During the process of establishing the model, we stepwise adjusted for confounding factors. Model 1 represents a crude model without any variable adjustments. In Model 2, age and gender were adjusted, while in Model 3, covariates were fully adjusted, including age, gender, race, education level, PIR, smoking status, alcohol intake, and BMI. All data were extracted and analyzed using the R software (version 4.3.0), and *p* < 0.05 was defined as statistically significant.

## Results

### Characteristics of the participants

The study collected data from the National Health and Nutrition Examination Survey (NHANES) 2007–2018. A total of 25,801 US adults with dietary selenium intake information and mortality data were included in the analysis. Table 1 shows the baseline characteristics of the eligible participants. The mean age of these individuals was 47 ± 17 years, and 49% were male. Non-Hispanic White was the most common race in this research (68%). Among the subjects, people suffering from hypertension, diabetes, and CVD accounted for 32, 9.8, and 8.6%, respectively. Selenium intake was classified into quartiles (Q1–Q5) based on the values of two 24-h dietary interviews. Compared with Q1, the participants in Q2–Q5 tended to have higher levels of education and PIR, there were fewer smokers among them, and they were more likely to drink alcohol. There was no significant difference in body mass index (BMI) and cancer morbidity between the groups.

**Table 1:** Characteristics of the participants in this study stratified by selenium intake.

Dietary selenium intake (quintile)							
Characteristic	Overall, *N* = 25,801 (100%)^2^	Q1, *N* = 5,680 (20%)^2^	Q2, *N* = 5,363 (20%)^2^	Q3, *N* = 5,082 (20%)^2^	Q4, *N* = 4,996 (20%)^2^	Q5, *N* = 4,680 (20%)^2^	*p*-value^3^
**Age (years)**	47 [47 (17)]	48 [48 (18)]	47 [48 (17)]	47 [48 (17)]	46 [47 (17)]	47 [47 (16)]	0.005
**Gender (%)**							<0.001
*Female*	13,009 (51%)	4,022 (75%)	3,269 (63%)	2,571 (53%)	2,018 (42%)	1,129 (24%)	
*Male*	12,792 (49%)	1,658 (25%)	2,094 (37%)	2,511 (47%)	2,978 (58%)	3,551 (76%)	
**Race (%)**							<0.001
*Mexican American*	3,745 (8.2%)	826 (7.7%)	795 (8.7%)	732 (7.9%)	722 (8.4%)	670 (8.1%)	
*Other Hispanic*	2,551 (5.4%)	617 (6.0%)	542 (5.4%)	522 (5.5%)	474 (5.4%)	396 (4.8%)	
*Non-Hispanic White*	11,354 (68%)	2,310 (66%)	2,343 (68%)	2,260 (69%)	2,271 (69%)	2,170 (70%)	
*Non-Hispanic Black*	5,424 (11%)	1,454 (14%)	1,159 (11%)	1,008 (10%)	942 (9.6%)	861 (8.5%)	
*Other*	2,727 (7.4%)	473 (6.5%)	524 (6.9%)	560 (7.7%)	587 (7.9%)	583 (8.2%)	
**Educational level (%)**							<0.001
*≤High school*	5,879 (15%)	1,678 (20%)	1,292 (15%)	1,050 (13%)	1,014 (13%)	845 (12%)	
*>High school*	19,922 (85%)	4,002 (80%)	4,071 (85%)	4,032 (87%)	3,982 (87%)	3,835 (88%)	
**PIR (%)**							<0.001
*< 1.3*	8,138 (22%)	2,194 (28%)	1,806 (25%)	1,499 (20%)	1,469 (21%)	1,170 (17%)	
*≥3.5*	7,868 (43%)	1,288 (35%)	1,521 (40%)	1,626 (46%)	1,677 (45%)	1,756 (51%)	
*1.3–3.5*	9,795 (35%)	2,198 (37%)	2,036 (36%)	1,957 (35%)	1,850 (35%)	1,754 (32%)	
**Smoke status (%)**							<0.001
*Current smoker*	5,346 (20%)	1,316 (25%)	1,097 (20%)	988 (19%)	990 (19%)	955 (18%)	
*Former smoker*	6,421 (25%)	1,211 (21%)	1,262 (23%)	1,279 (26%)	1,346 (28%)	1,323 (28%)	
*Never smoker*	14,034 (55%)	3,153 (55%)	3,004 (56%)	2,815 (56%)	2,660 (53%)	2,402 (54%)	
**Alcohol intake (%)**							<0.001
*Drinker*	17,819 (74%)	3,407 (66%)	3,512 (71%)	3,588 (75%)	3,648 (77%)	3,664 (82%)	
*Never drinker*	7,982 (26%)	2,273 (34%)	1,851 (29%)	1,494 (25%)	1,348 (23%)	1,016 (18%)	
**BMI group (%)**							0.2
*Normal*	6,768 (26%)	1,426 (24%)	1,390 (26%)	1,362 (26%)	1,333 (27%)	1,257 (27%)	
*Obese*	10,323 (40%)	2,325 (41%)	2,196 (41%)	1,951 (39%)	1,973 (39%)	1,878 (40%)	
*Overweight*	8,325 (32%)	1,824 (32%)	1,693 (31%)	1,716 (34%)	1,600 (32%)	1,492 (32%)	
*Underweight*	385 (1.5%)	105 (1.8%)	84 (1.5%)	53 (1.2%)	90 (1.8%)	53 (1.1%)	
**Hypertension (%)**							0.2
*No*	16,356 (68%)	3,435 (67%)	3,366 (67%)	3,257 (69%)	3,270 (69%)	3,028 (67%)	
*Yes*	9,445 (32%)	2,245 (33%)	1,997 (33%)	1,825 (31%)	1,726 (31%)	1,652 (33%)	
**Cardiovascular disease (%)**							<0.001
*No*	22,972 (91%)	4,901 (89%)	4,765 (91%)	4,550 (92%)	4,519 (93%)	4,237 (92%)	
*Yes*	2,829 (8.6%)	779 (11%)	598 (8.8%)	532 (8.3%)	477 (7.3%)	443 (8.1%)	
**Diabetes (%)**							0.037
*No*	22,384 (90%)	4,814 (89%)	4,615 (89%)	4,441 (91%)	4,395 (91%)	4,119 (91%)	
*Yes*	3,417 (9.8%)	866 (11%)	748 (11%)	641 (9.3%)	601 (9.3%)	561 (8.9%)	
**Cancer (%)**							0.6
*No*	23,223 (89%)	5,082 (89%)	4,796 (89%)	4,604 (90%)	4,505 (90%)	4,236 (90%)	
*Yes*	2,578 (11%)	598 (11%)	567 (11%)	478 (10%)	491 (10%)	444 (10%)	

Q1, quintile 1; Q2–Q5, higher quintiles; PIR, poverty index; BMI, body mass index. ^2^The percentage of participates with different quintiles of selenium intake to the total number of participants. ^3^The *p*-value of Wilcoxon rank sum test and Kruskal Wallis test for calculating baseline characteristic differences between populations with different quintiles of selenium intake.

### Association between selenium intake and human chronic disease

A multivariate logistic regression model was created to evaluate the relationship between selenium intake and human chronic diseases, such as CVD, DM, and cancer (Table 2). In the crude model, compared with Q1, the risk of CVD decreased in participants with higher quintiles (Q2–Q5) of selenium consumption (with OR = 0.98, 95% CI: 0.97–1.00, *p* = 0.022; OR = 0.98, 95% CI: 0.96–0.99, *p* = 0.008; OR = 0.97, 95% CI: 0.95–0.98, *p* < 0.001; OR = 0.98, 95% CI: 0.96–0.99, *p* = 0.005, respectively). In Model 2, adjusted for age and gender, the OR of CVD still decreased with increasing selenium intake (the ORs for Q1–Q4 are 0.98, 95% CI: 0.97–1.00, *p* = 0.025; 0.98, 95% CI: 0.96–0.99, *p* = 0.008; 0.97, 95% CI: 0.95–0.98, *p* < 0.001 and 0.97, 95% CI: 0.95–0.99, *p* < 0.001, respectively). In addition, the results of the logistic regression analysis showed that the risk of DM decreased when selenium intake reached 0.12920 (g) and above (Q4 and Q5), regardless of age and gender adjustment (the ORs for Q4 and Q5 in Model 1 are 0.98, 95% CI: 0.97–1.00, *p* = 0.047; 0.98, 95% CI: 0.97–1.00, *p* = 0.009, respectively, and in Model 2 are 0.98, 95% CI: 0.97–1.00, *p* = 0.039; 0.98, 95% CI: 0.96–0.99, *p* = 0.001, respectively). After adjusting all the possible confounders (Model 3), Q4 and Q5 of selenium intake still had a protective effect on preventing the occurrence of CVD. However, selenium supplementation did not show a statistically significant reduction in OR for DM when all of the suspected covariates were adjusted. Moreover, there was no statistically significant correlation between any level of selenium consumption and cancer risk.

**Table 2:** The association between dietary selenium intake and the risk of CVD, TDM and cancer.

Dietary selenium intake (quintile)	Model 1			Model 2			Model 3		
	Exp (Beta)	95% CI^1^	*p*-value	Exp (Beta)	95% CI^1^	*p*-value	Exp (Beta)	95% CI^1^	*p*-value
**The risk of CVD**									
Q1	—	—		—	—		—	—	
Q2	0.98	0.97, 1.00	0.022	0.98	0.97, 1.00	0.025	0.99	0.97, 1.00	0.11
Q3	0.98	0.96, 0.99	0.008	0.98	0.96, 0.99	0.008	0.99	0.97, 1.00	0.10
Q4	0.97	0.95, 0.98	<0.001	0.97	0.95, 0.98	<0.001	0.97	0.96, 0.99	0.002
Q5	0.98	0.96, 0.99	0.005	0.97	0.95, 0.99	<0.001	0.98	0.97, 1.00	0.038
**The risk of TDM**									
Q1	—	—		—	—		—	—	
Q2	0.99	0.98, 1.01	0.4	1.00	0.99, 1.01	>0.9	1.00	0.98, 1.01	0.7
Q3	0.99	0.97, 1.01	0.2	1.00	0.99, 1.02	0.8	1.00	0.98, 1.01	0.6
Q4	0.99	0.97, 1.00	0.13	1.00	0.99, 1.02	0.5	1.00	0.98, 1.01	0.9
Q5	0.99	0.97, 1.01	0.2	1.01	0.99, 1.03	0.2	1.00	0.99, 1.02	0.8
**The risk of cancer**									
Q1	—	—		—	—		—	—	
Q2	1.00	0.98, 1.01	0.7	1.00	0.98, 1.01	0.8	1.00	0.99, 1.02	0.7
Q3	0.98	0.97, 1.00	0.060	0.98	0.97, 1.00	0.056	0.99	0.98, 1.01	0.4
Q4	0.98	0.97, 1.00	0.047	0.98	0.97, 1.00	0.039	0.99	0.98, 1.01	0.3
Q5	0.98	0.97, 1.00	0.009	0.98	0.96, 0.99	0.001	0.99	0.97, 1.00	0.10

Model 1: crude model. Model 2: adjusted for age and gender. Model 3: adjusted for age, gender (male and female), race (non-Hispanic white, non-Hispanic black, Mexican American, Other Hispanic, or other ethnicity), educational level (≤high school, or >high school), family poverty income ratio (<1.3, 1.3–3.5, or >3.5), smoking status (current smoker, former smoker, or never smoker), alcohol intake (drinker, or never drinker), body mass index (underweight, normal overweight, or obese). ^1^CI, confidence interval; Q1, quintile 1; Q2–Q5, higher quintiles; CVD, cardiovascular disease; TDM, Diabetes mellitus.

### Association between selenium intake with all-cause and disease-specific mortality

The participants in this study had a median follow-up period of 81 months, and a total of 2,436 deaths events were recorded at the endpoint. As shown in Table 3, the association between selenium intake and the risk of all-cause and disease-specific mortality was calculated based on a multivariate Cox proportional hazard model. The dietary selenium intake showed a significant negative correlation with the all-cause mortality risk in the unadjusted model (Model 1). Compared with Q1 (reference), Q2–Q5 of selenium intake were linked with reduced mortality risk, with all *p*-values <0.05. These correlations also were observed in Model 2 (adjusting for age and race). The results of Model 3 (adjusting for all covariates) indicated that Q5 selenium intake was significantly correlated with a lower risk of all-cause mortality (HR = 0.82, 95% CI: 0.68–0.99, *p* = 0.037). After adjusting for all covariates, the disease-specific mortality results indicated that moderate to high levels of selenium intake were associated with decreased risk of CVD and T2DM-specific death. In detail, the participants with Q4 selenium intake had a lower HR for CVD-specific mortality compared with the participants in Q1 (HR = 0.40, 95% CI: 0.16–0.97, *p* = 0.043). Moreover, the DM-specific HR was decreased in participants with Q2–Q4 of selenium (HR = 0.30, 95% CI: 0.12–0.75, *p* = 0.011; HR = 0.44, 95% CI: 0.21–0.94, *p* = 0.035; HR = 0.36, 95% CI: 0.14–0.92, *p* = 0.033, respectively). On the contrary, there was no significant correlation among the different groups in the crude model of CVD and DM mortality analysis. This may be attributed to smoking, alcohol consumption, and BMI, which are considered important risk factors for accelerating the progression of CVD and DM. After adjusting for these variables, the protective effect of selenium on disease-specific mortality can be more clearly demonstrated. However, there were no significant associations between selenium intake and cancer-related mortality in either the original model or the model adjusted for confounding factors.

**Table 3:** Association between selenium intake with all-cause and disease specific mortality.

Dietary selenium intake (quintile)	Model 1			Model 2			Model 3		
	HR^1^	95% CI^2^	*p*-value	HR^1^	95% CI^2^	*p*-value	HR^1^	95% CI^2^	*p*-value
**All-cause mortality**									
Q1	—	—		—	—		—	—	
Q2	0.83	0.70, 0.99	0.035	0.83	0.71, 0.96	0.013	0.86	0.73, 1.00	0.056
Q3	0.74	0.63, 0.88	<0.001	0.74	0.63, 0.88	<0.001	0.85	0.71, 1.01	0.070
Q4	0.79	0.66, 0.93	0.006	0.80	0.68, 0.95	0.013	0.92	0.78, 1.09	0.3
Q5	0.67	0.56, 0.79	<0.001	0.68	0.57, 0.81	<0.001	0.82	0.68, 0.99	0.037
**CVD-specific mortality**									
Q1	—	—		—	—		—	—	
Q2	1.51	0.78, 2.90	0.2	1.42	0.77, 2.62	0.3	1.10	0.45, 2.66	0.8
Q3	0.77	0.37, 1.59	0.5	0.76	0.36, 1.59	0.5	0.76	0.38, 1.50	0.4
Q4	0.59	0.26, 1.33	0.2	0.58	0.24, 1.42	0.2	0.40	0.16, 0.97	0.043
Q5	1.45	0.88, 2.38	0.14	1.36	0.70, 2.63	0.4	1.21	0.66, 2.23	0.5
**TDM-specific mortality**									
Q1	—	—		—	—		—	—	
Q2	0.52	0.25, 1.07	0.075	0.46	0.22, 0.96	0.039	0.30	0.12, 0.75	0.011
Q3	1.02	0.55, 1.90	>0.9	0.95	0.51, 1.77	0.9	0.44	0.21, 0.94	0.035
Q4	0.82	0.45, 1.50	0.5	0.79	0.44, 1.42	0.4	0.36	0.14, 0.92	0.033
Q5	0.58	0.31, 1.10	0.10	0.51	0.23, 1.14	0.10	0.52	0.17, 1.60	0.3
**Cancer-specific mortality**									
Q1	—	—		—	—		—	—	
Q2	0.99	0.65, 1.51	>0.9	0.95	0.64, 1.42	0.8	0.95	0.66, 1.38	0.8
Q3	0.99	0.69, 1.41	>0.9	0.96	0.70, 1.34	0.8	0.94	0.68, 1.29	0.7
Q4	0.87	0.64, 1.19	0.4	0.88	0.66, 1.17	0.4	0.91	0.68, 1.22	0.5
Q5	1.06	0.72, 1.57	0.8	1.01	0.69, 1.47	>0.9	1.05	0.71, 1.55	0.8

Model 1: crude model. Model 2: adjusted for age and sex. Model 3: adjusted for age, sex (male and female), race (non-Hispanic white, non-Hispanic black, Mexican American, Other Hispanic, or other ethnicity), educational level (≤ high school, or >high school), family poverty income ratio (<1.3, 1.3–3.5, or >3.5), smoking status (current smoker, former smoker, or never smoker), alcohol intake (drinker, or never drinker), body mass index (underweight, normal overweight, or obese). ^1^HR, hazard ratio; ^2^CI, confidence interval; Q1, quintile 1; Q2–Q5, higher quintiles; CVD, cardiovascular disease; TDM, Diabetes mellitus.

**Figure 2:**
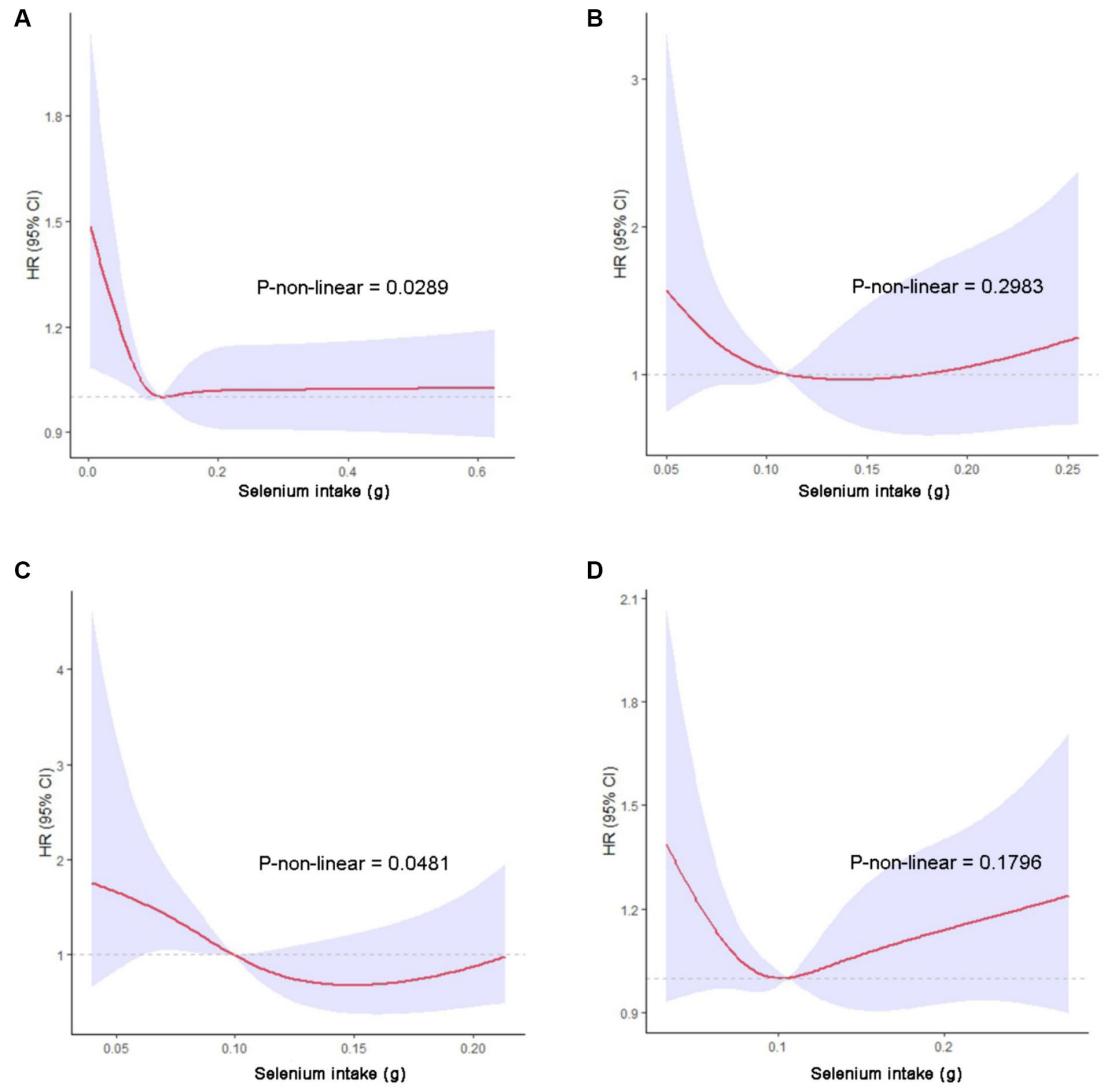
The RCS analysis between selenium intake and all-cause, CVD-specific, DM-specific and cancer-specific mortality. The red line represents hazard ratio, and blue area represent the 95% confidence interval of estimated HR. **(A)** All-cause mortality; **(B)** CVD-specific mortality; **(C)** DM-specific mortality; **(D)** cancer-specific mortality.

We performed a restricted cubic spline (RCS) to further illustrate the relationship between selenium intake and all-cause and cause-specific mortality. The results of the RCS analysis based on Cox regression models indicated that selenium had a statistically significant nonlinear correlation with all-cause and DM-specific death (*p* for nonlinearity in all-cause mortality = 0.0289; *p* for nonlinearity in diabetes-specific mortality = 0.0481). After multivariate adjustment, the RCS curves presented different dose-response relationships depending on the cause of death (Figure 2).

### Subgroup analysis

**Figure 3:**
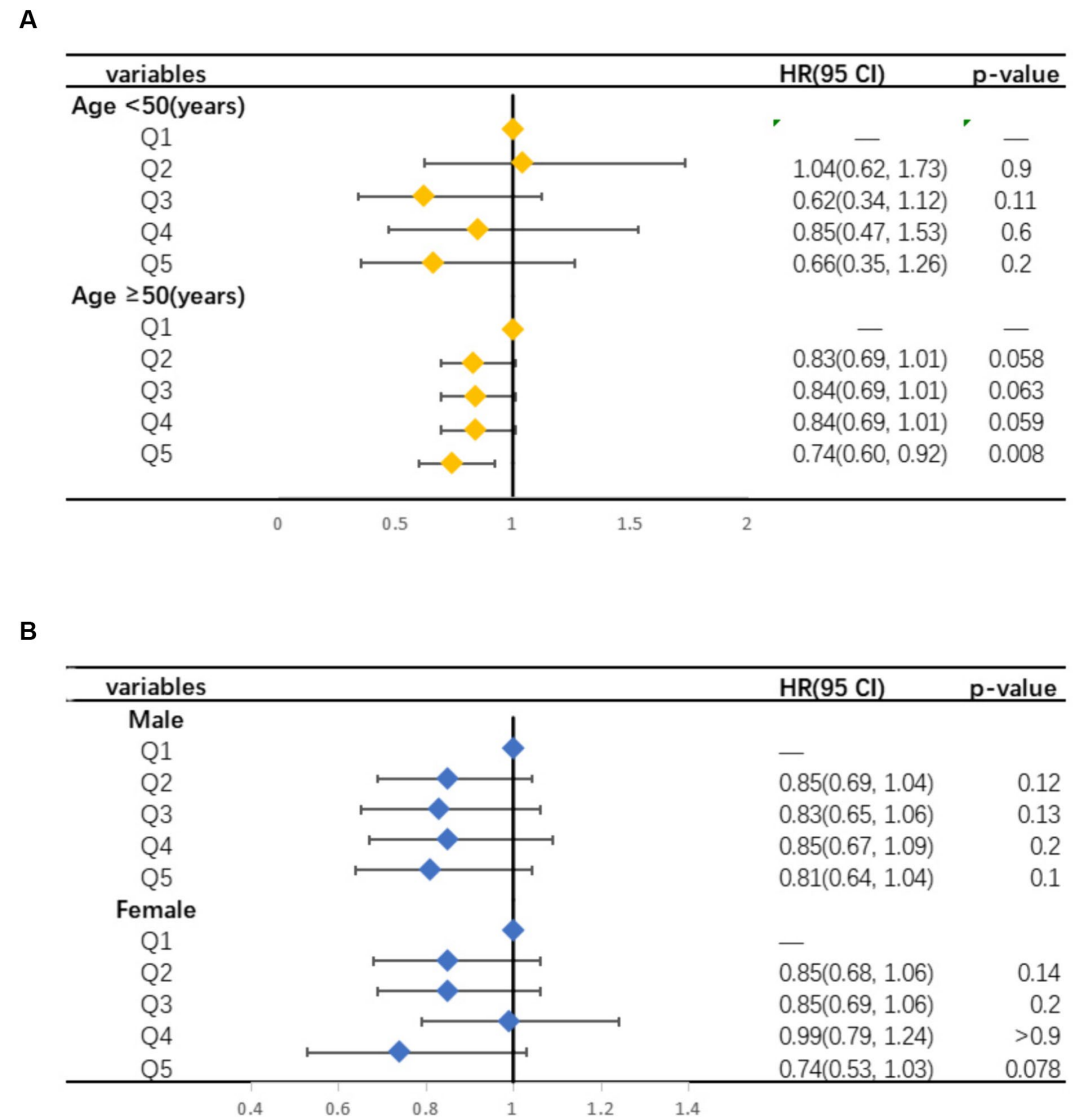
Forest plot of the associations between selenium intake and the risk of all-cause mortality as age-stratified **(A)** and gender-stratified **(B)**. The value of ORs was calculated using multivariable Cox proportional hazards regression.

Finally, we investigated the relationships in the subgroups divided by age and sex. A significant association was observed between the highest quartile of selenium intake and all-cause mortality among participants aged 50 and above (HR = 0.75, 95% CI: 0.60–0.93, *p* = 0.009). No statistical interactions were found between the different quintiles of selenium intake and the risk of mortality among people younger than 50 years. Different genders have no effect on the relationship between selenium intake and all-cause mortality (Table 4 and Figure 3).

**Table 4:** Subgroup analyses.

Dietary selenium intake (quintile)	Age <50			Age ≥50		
	HR^1^	95% CI^2^	*p*-value	HR^1^	95% CI^2^	*p*-value
Q1	—	—		—	—	
Q2	1.04	0.62, 1.73	0.9	0.83	0.69, 1.01	0.058
Q3	0.62	0.34, 1.12	0.11	0.84	0.69, 1.01	0.063
Q4	0.85	0.47, 1.53	0.6	0.84	0.69, 1.01	0.059
Q5	0.66	0.35, 1.26	0.2	0.74	0.60, 0.92	0.008

Dietary selenium intake (quintile)	Male			Female		
	HR^1^	95% CI^2^	*p*-value	HR^1^	95% CI^2^	*p*-value
Q1	—	—		—	—	
Q2	0.85	0.69, 1.04	0.12	0.85	0.68, 1.06	0.14
Q3	0.83	0.65, 1.06	0.13	0.85	0.69, 1.06	0.2
Q4	0.85	0.67, 1.09	0.2	0.99	0.79, 1.24	>0.9
Q5	0.81	0.64, 1.04	0.10	0.74	0.53, 1.03	0.078

Subgroup analysis of the association between selenium intake (classification) and all-cause mortality. Adjusted for age, sex, race, educational level, family poverty income ratio, smoking status, alcohol intake and body mass index.^1^HR, hazard ratio; ^2^CI, confidence interval.

## Discussion

Although several studies have been conducted on the relationship between selenium intake and human health, this study has a very high sample size. In this retrospective study based on the NHANES database, we noted a negative correlation between higher levels of dietary selenium intake and the morbidity of CVD. However, this relationship was not significant in T2DM and cancer. Furthermore, our research also found that selenium intake was nonlinearly associated with all-cause mortality, CVD-related mortality, and T2DM-related mortality after multivariable adjustment. These findings provide a theoretical basis for reducing the incidence of CVD and mortality by moderate dietary selenium intake.

As an essential micronutrient, selenium has always been considered to play an important role in decreasing the occurrence of CVD. As early as 1979, the results of an epidemiological study reported that selenium deficiency is one of the causes of the Keshan disease, marking the first time humans noticed the relationship between selenium deficiency and human diseases (28). In recent decades, several prospective studies have generally shown a significant negative correlation between dietary selenium intake or serum selenium levels and the greater protection of CVD (29–31). Selenium and selenoprotein mainly block the development of CVD by inhibiting the inflammatory response of the aortic endothelial cells, weakening ROS-induced oxidative stress, and preventing vascular calcification (32–34). In our present study, higher quintiles of dietary selenium intake resulted in the up-regulated risk of CVD, consistent with previous research. It is worth noting that after multivariate adjustment, higher levels of selenium intake were still associated with decreased ORs of CVD, indicating that selenium intake was an independent risk factor for CVD. Furthermore, the results from several randomized controlled trials and experimental studies have demonstrated that selenium consumption above 200 μg/day has a linear relationship with TDM incidence (35, 36). However, in our analysis, there was no statistically significant correlation between higher levels of selenium intake and the risk of TDM after covariate adjustment. This may be due to the differences in baseline characteristics among the participants included in different studies. Additionally, other studies have found that dietary selenium plays a role in preventing cancer incidence, especially colorectal cancer, prostatic cancer, and non-melanoma skin cancer (37–39). At present, some selenium-based compounds or selenium derivatives have shown excellent anticancer activity in experiments (40, 41). These studies provided a basis for the application of selenium in future cancer treatment and further elucidated the protective effects of selenium on preventing cancer occurrence. However, in our study, we did not draw a similar conclusion that selenium intake can reduce the morbidity of cancer, which may be attributed to the fact that we did not make a detailed distinction between cancer types.

Previous studies have proven that adequate selenium consumption promotes human health by improving metabolism, enhancing immunity, and delaying aging, which is strongly negatively correlated with all-cause mortality (13, 42, 43). In another study, Jenkins et al. (44) indicated that supplementing selenium helps to enhance the protective effect of antioxidant mixtures on cardiovascular disease and reduce all-cause mortality. Our findings also show that moderate to high levels of selenium intake reduces the risk of all-cause mortality. Some studies have shown that selenium can increase the expression and phosphorylation of endothelial nitric oxide synthase (eNOS), thereby protecting the vitality and migration ability of endothelial cells (45). Supplementing selenium and coenzyme Q10 can improve the systemic redox state and is significantly associated with a reduced risk of cardiovascular mortality in older individuals (46). According to the results of our Cox proportional hazards model, dietary selenium intake was nonlinearly correlated with all-cause mortality and CVD-related mortality, which is consistent with previous studies. The correlation between selenium intake and the occurrence or prognosis of diabetes has always been controversial. The results of a cross-sectional study from Wei et al. (47) showed that dietary selenium intake had a moderately negative correlation with diabetes related metabolic comorbidities (MetS). A recent research conducted by Kamali et al. (48) indicated that an appropriate selenium supplementation improved glucose metabolism by reducing fasting plasma glucose (FPG). While some studies even found that high selenium intake can promote insulin resistance. In this study, we observed that an appropriate higher selenium intake contributes to reducing diabetes-related mortality but has no effect on cancer-related mortality (after all covariate adjustments). Although several retrospective analyses have shown the protective effect of dietary selenium intake on cancer (especially digestive system cancers) (49), whereas its underlying mechanisms are still unclear, and further exploration is required.

To further verify the effect of selenium on all-cause mortality in different groups, we performed a subgroup analysis. The participants in this study were stratified by age and gender. As shown in Table 4, we found that among the individuals aged ≥50 years, the highest quintile of selenium intake was significantly correlated with a decrease in all-cause mortality. Part of the reason for this finding may be attributed to changes in the ability of older adults to utilize micronutrients. Previous studies have shown that older adult people are affected by factors such as chronic diseases and medications intake, leading to a decrease in their ability to utilize nutrition (50). Additionally, the distribution of selenium in plasma selenoproteins is also influenced by age (51). Blood selenium concentration is negatively correlated with the risk of malnutrition in older adults (52). Insufficient selenium intake may affect physical activity and self-awareness, thereby influencing the quality of life of older individuals (53). Therefore, we speculate that older adult people consuming selenium rich diets such as fish and meat may have better protective effects on health, but this hypothesis still needs further experimental verification. Meanwhile, the effect of selenium intake on all-cause mortality was similar in males and females. In summary, the results of our subgroup analysis indicated that older adults may benefit better from dietary selenium supplementation, providing a theoretical basis for rational selenium supplementation for US adults, especially the older population. In addition, we have noticed that some studies suggest a risk of selenium deficiency in the daily diet of infants and preschool children, which seriously endangers their growth, development, and health (54). However, currently epidemiological studies lack of dietary selenium intake data of infants and preschool children. Therefore, it is necessary to comprehensively analyze the selenium intake status of children in different age groups in the future, and provide nutritional recommendations for whether selenium supplement is needed in the children’s diets.

Selenium has a wide range of sources in our daily diet, including meat (especially beef, pork and fish) (55, 56), nuts (57), and grains (58). In addition, some multivitamins, such as Australian multivitamins (Elevit) and microbial supplements, are also part of the dietary selenium source (59). Environment and dietary habits are important factors affecting human selenium intake. Long-term vegetarianism or living in low-selenium areas may lead to selenium deficiency in human body (20, 60). The World Health Organization (WHO) recommends an intake of 55–60 μg of selenium per day (61). In this study, our results demonstrate that selenium intake ≥0.129 μg/day may be a protective factor in reducing all-cause and cardiovascular disease-related mortality, consistent with previous studies. However, excessive consumption of selenium is proven to be poisonous (62, 63). Therefore, it is not recommended for the general population to take additional selenium supplements to prevent diseases.

There are several advantages of this study. First, we collected information from the NHANES database covering six cycles and ultimately included 25,801 individuals, making our data in this analysis large and representative. Second, we divided selenium intake in diet and dietary supplements into quintiles and provided a detailed dose-response relationship between selenium intake and all-cause or disease-specific mortality by plotting the RCS curves. Furthermore, given the controversy over the impact of selenium intake on diabetes in previous research, this study explored the relationship between the two using a large sample of people and found that higher levels of selenium intake can reduce the risk of diabetes-specific mortality. Although the potential mechanisms of this conclusion still need to be explored, our research undoubtedly provides a new perspective for dietary intervention in diabetes patients. Finally, this study systematically analyzed the impact of selenium intake on the occurrence, development, and prognosis of three major chronic diseases (CVD, TDM and cancer) in humans, emphasizing the importance of moderate selenium intake in diet and dietary supplements for maintaining human health.

Admittedly, our study also has several deficiencies. First, this is a retrospective study, and the research findings may have been influenced by unmeasured and unnoticed biases and confounding factors, which cannot be completely avoided despite complex statistical adjustments. Second, the dietary data in this study are sourced from 24-h telephone recalls and do not represent the long-term selenium intake of the participants. Therefore, the conclusions of this study need to be carefully interpreted. Third, when exploring the correlation between selenium intake and the occurrence or prognosis of cancer, we did not provide a detailed classification of cancer types. Cancer is a highly heterogeneous disease (64, 65); thus, our conclusion may dilute the relationship between the two. Finally, the participants in this study are adults in the US. In the future, we need to further explore whether these conclusions are applicable to other populations, especially children.

## Conclusion

The findings suggest that moderate to high levels of selenium (≥ 0.129 μg/day) intake are associated with decreased risk for CVD incidence. In addition, appropriate selenium intake may be a protective factor in reducing the risk of all-cause and CVD-related mortality. Therefore, dietary intervention based on the consumption of a healthy diet rich in selenium is a promising measure for maintaining health and prolonging survival time for US adults.

## Data availability statement

The datasets presented in this study can be found in online repositories. The names of the repository/repositories and accession number(s) can be found at: https://www.cdc.gov/nchs/nhanes/index.htm.

## Ethics statement

The studies involving humans were approved by the National Center for Health Statistics, CDC. The studies were conducted in accordance with the local legislation and institutional requirements. Written informed consent for participation was not required from the participants or the participants’ legal guardians/next of kin in accordance with the national legislation and institutional requirements.

## Author contributions

YZ: Formal analysis, Methodology, Writing – original draft. SM: Methodology, Software, Visualization, Writing – original draft. YY: Formal analysis, Investigation, Writing – review & editing. LB: Methodology, Writing – review & editing. JT: Conceptualization, Formal analysis, Investigation, Writing – review & editing. LZ: Supervision, Validation, Writing – review & editing.
